# Initial lactate levels linked to oliguria in term neonates with perinatal asphyxia

**DOI:** 10.1007/s00467-024-06322-8

**Published:** 2024-02-27

**Authors:** Yu Zhang, Huihui Zeng

**Affiliations:** Neonatal Intensive Care Unit, Beijing Obstetrics and Gynecology Hospital, Capital Medical University. Beijing Maternal and Child Health Care Hospital, Beijing, China

**Keywords:** Oliguria, Asphyxia, Lactate, Arterial blood gas, Acute kidney injury, Neonates

## Abstract

**Background:**

Oliguria is a sign of impaired kidney function and has been shown to be an early predictor of adverse prognoses in patients with acute kidney injury. The relationship between urine output (UOP) and early lactate levels in neonates with perinatal asphyxia (PA) has not been extensively explored. This study aimed to investigate the link between oliguria during the first 24 h of life and early lactate levels in neonates with PA.

**Methods:**

The medical records of 293 term neonates with asphyxia from 9216 hospitalized newborns were retrospectively analyzed, including 127 cases designated as the oliguria group and 166 cases as controls. Peripheral arterial blood gas after PA and UOP within 24 h after birth were analyzed. Logistic regression analyses and receiver operating characteristic curve analysis were conducted.

**Results:**

Oliguria occurred in 43.34% of neonates with PA. The median UOP of the oliguria and control groups were 0.65 and 1.46 mL/kg/h, respectively. Elevated lactate levels after PA are an independent risk factor for oliguria in the following 24 h (p = 0.01; OR: 1.19; 95%CI: 1.04–1.35) and show a moderate discriminatory power for oliguria (AUC = 0.62). Using a cut off value of 8.15 mmol/L, the positive and negative predictive values and the specificity were 59.34%, 63.86%, and 78.30%, respectively.

**Conclusion:**

Neonates with elevated lactate levels after PA face a risk of oliguria in the following 24 h. Based on early elevated lactate levels after resuscitation, especially ≥ 8.15 mmol/L, meticulously monitoring UOP will allow this vulnerable population to receive early, tailored fluid management and medical intervention.

**Graphical abstract:**

**Figure:**
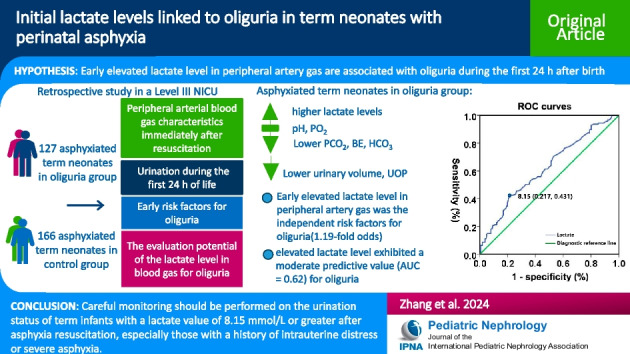
A higher resolution version of the Graphical abstract is available as Supplementary information

**Supplementary Information:**

The online version contains supplementary material available at 10.1007/s00467-024-06322-8.

## Introduction 

Acute kidney injury (AKI) is associated with longer hospital stays and increased morbidity and mortality in critically ill adult, pediatric, and neonatal populations [1, 2]. In neonates, urine output (UOP) criteria have been utilized as one of multiple definitions of AKI during the past decade [3]. The neonatal Risk, Injury, Failure, Loss, and End-stage renal disease (RIFLE) classification (2013) includes UOP criteria without creatinine criteria [4]. Neonates with a UOP lower than 1.5 mL/kg/h for 24 h are included in the AKI risk observation population [4]. In the more commonly used AKI definition (Neonatal Modification 2016) by Kidney Disease: Improving Global Outcomes (KDIGO), the stage 1 definition involves a UOP threshold value of less than 1 ml/kg per hour, and the stage 2 definition involves a UOP threshold value of less than 0.5 ml/kg per hour for 24 h [5, 6]. Currently, the UOP threshold in the neonatal population, especially on day of life 1, is not known. Urine volume is a vital and noninvasive clinical indicator especially in the early neonatal stages. Oliguria is not only a sign of impaired kidney function but also an inexpensive and impactful predictive biomarker of AKI in critically ill patients [1, 3, 7]. Additionally, oliguria has been shown to be an early predictor of adverse prognoses or increased mortality in survivors [7, 8].

During perinatal asphyxia (PA), hemodynamic disturbances can cause varying degrees of injury to vital organs, such as the brain, heart, lungs, kidneys and liver [9]. Among full-term neonates with PA, the kidney is the most affected organ [10], being very sensitive to PA and renal insufficiency occurring within 24 h of hypoxia and ischemia [10]. Full-term neonates with asphyxia have a high incidence of AKI, which can be as high as 50–72% [11]. For vulnerable neonates, early evaluation of oliguria risk in term neonates with asphyxia within 24 h of life can provide useful information for timely intervention or treatment, including fluid and electrolyte management, drug choice, such as antibiotic dosage choice and parenteral nutrition. We have been striving to find an easily accessible clinical factor that can indicate the UOP and predict oliguria in newborns with asphyxia.

At our center, for all neonates with asphyxia, arterial blood gas analysis is required immediately after admission to help develop a post-resuscitation treatment plan, and reports are available as early as half an hour to an hour after birth. Blood gas analysis and lactate values, reflecting decreased oxygen levels and acidosis, are widely used to assess neonatal ischemic encephalopathy, critical management, and death [11–13]. However, the relationship between UOP for 24 h and early radial artery blood gas analysis and lactate levels in neonates with PA has not been extensively explored. Based on the need for optimal monitoring of neonates after asphyxia, this study was conducted to preliminarily explore the early prediction value of peripheral arterial blood gas results obtained soon after birth in detecting the oliguria risk in full-term neonates with asphyxia after resuscitation on the first day of life.

## Materials and methods

### Study design and population

After obtaining ethical clearance (2023-KY-014–01), this preliminary retrospective study was conducted in the NICU of a level 3 hospital in Beijing between January 2019 and December 2022. Initially, all full-term neonates with asphyxia (gestational age ≥ 37 weeks and < 42 weeks) from 9216 hospitalized newborns were retrospectively analyzed, and 296 full-term neonates with asphyxia hospitalized for more than 1 day in the NICU were included. After applying the exclusion criteria, 293 full-term neonates with asphyxia were selected for this study, with 127 cases designated to the oliguria group and 166 cases to the control group (those with normal UOP). The exclusion criteria were as follows: (a) congenital deformity, (b) inherited metabolic disease, (c) family history of kidney disease, (d) inappropriate fluid and electrolyte management as per our NICU protocol, (e) use of mannitol, ibuprofen or diuretic within 24 h after birth, (f) lack of arterial blood gas record or continuous diagnosis and treatment data; (g) renal dysfunction in mothers during the perinatal period, (h) maternal use of drugs that affect kidney function during the perinatal period, and (i) mother experiencing severe anemia during the perinatal period.

Oliguria was defined as a UOP < 1 ml/kg/h during the first 24 h after birth [14, 15]. Perinatal asphyxia was defined according to the recommendations for the diagnosis and grading criteria of neonatal asphyxia in China [16] as follows: (a) high-risk factors that may cause suffocation before delivery, (b) Apgar score of 7 or less at 1 or 5 min, with effective spontaneous breathing not yet established, (c) umbilical artery blood pH value less than 7.15, and (d) excluding other causes of low Apgar scores. Severe perinatal asphyxia was defined based on an Apgar score of 3 or less at 1 min [16]. AKI was defined according to the serum creatinine criteria (Neonatal Modification 2016) proposed by KDIGO [5, 6]. Intrauterine distress is a respiratory and circulatory dysfunction syndrome caused by fetal hypoxia; it refers to the presence of signs of hypoxia in the uterus that endanger fetal health and life [17, 18]. Maternal complications recorded in this study included hypertensive disorder, diabetes, hypothyroidism, hemolysis, placental abruption, and intrapartum hemorrhage. The threshold value for lactate levels was derived from the ROC–AUC analysis and was used to divide the cohort into risk levels for oliguria.

### Data collection

Resuscitation, emergency care, and stabilization were prioritized over other operations. An experienced neonatology team uniformly managed all neonates included in this study according to standard NICU guidelines with guaranteed standard input. Radial artery blood gas analysis samples were collected as routine admission examination within 0.5 to 1 h after birth and analyzed at the local hospital laboratory (GEM premier 4000, Bedford, MA, USA). Blood gas analysis values, including pH, PCO_2_, partial pressure of oxygen (PO_2_), bicarbonate radical (HCO_3_^−^), base excess (BE), and lactic acid values, were transcribed from the medical records of hospitalized neonates. The neonates’ sex, birth weight, birth length, degree of asphyxia, fluid intake, urinary volume, and UOP within 24 h after birth were transcribed from the nursing records.

UOP was routinely measured by weighing the diaper every 3 h. The mothers’ information, including delivery mode, primiparity, perinatal complications, premature rupture of membranes, and fetal distress history, were transcribed from their medical records.

### Sample size calculation

No prior sample sizes were calculated. Neonates eligible during the study period were included in the study.

### Statistical analysis

Statistical analyses were conducted using SPSS (version 21.0; IBM Corp., Armonk, NY, USA). The significance level was set at α = 0.05. Measurement data distribution was presented using the median and interquartile range (IQR). Dichotomous data were described using absolute frequency (composition ratio). Non-normally distributed measurement data (birth weight or length, fluid intake, fluid intake/birth weight, urinary volume, UOP, lactate, pH, PCO_2_, PO_2_, BE, and HCO_3_^−^) were compared using the Mann–Whitney U test. Dichotomous data (primiparity, delivery mode, gestational complications, placental abruption, fetal distress, premature rupture of membranes, neonatal sex, severe perinatal asphyxia, oliguria, AKI, and mortality) were compared using the Chi-square or Fisher’s exact test. These comparisons were made between oliguria and control groups or between neonates with elevated and lower lactate levels. Potential risks and independent risk factors related to peripheral arterial blood gas for oliguria within the first 24 h of life in full-term neonates with PA were explored separately using univariate and multivariate logistic regression analyses. Additionally, receiver operating characteristic (ROC) curve analysis was conducted to investigate the estimated potential of lactate levels in blood gas analysis to predict oliguria.

## Results

### Study population

**Fig. 1:**
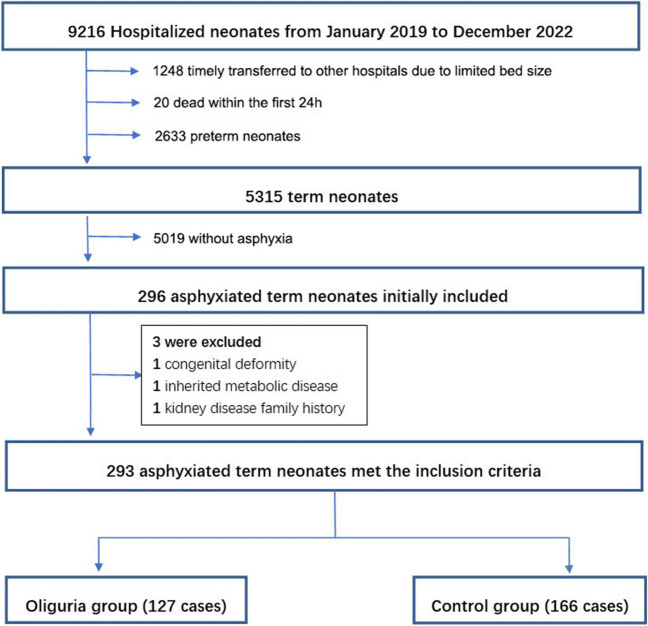
Study flow diagram. A total of 293 term neonates with asphyxia and a gestational age of 37 to 41 weeks were selected for the study and divided into two groups based on their urinary output within the first 24 h after birth

Between January 2019 and December 2022, our hospital’s neonatal intensive care unit treated 9216 hospitalized neonates. Initially, 296 full-term neonates with asphyxia hospitalized for more than 1 d in the NICU were included in the study. However, three were excluded for several reasons (Fig. 1). Ultimately, 293 full-term neonates with asphyxia and a gestational age of 37–41 weeks were included in the study, with 127 in the oliguria group and 166 in the control group.

The clinical characteristics of the term neonates with asphyxia and their mothers are presented in Table 1. There were no significant differences in sex, birth weight or length, fluid intake, or severe perinatal asphyxia occurrence between neonates in the oliguria and control groups (p > 0.05). There were no significant differences in the occurrence of primiparity, placental abruption, fetal distress, premature rupture of membranes, or gestational complications between the oliguria and control groups. The rate of vaginal delivery was significantly higher in the oliguria group than in the control group (95/127 (74.80%) vs. 85/166 (51.20%); χ^2^ = 16.91, p < 0.01)). We presumed that newborns delivered via cesarean section have a higher fluid load due to not being compressed through the birth canal and undergoing a shorter delivery process. Oliguria occurred in 43.34% (127/293) of neonates with PA. The urinary volume (50.00 vs. 116.00 mL; z = –14.14, p < 0.01) and UOP (0.65 vs. 1.46 mL/kg/h; z = –14.67, p < 0.01) in the oliguria group were significantly lower than those in the control group.

**Table 1:** Clinical characteristics of asphyxiated term neonates with oliguria and their mothers

Variables			UOP < 1.0 ml/kg/h (n = 127)	UOP ≥ 1.0 ml/kg/h (n = 166)	*p*- value
Maternal baseline	Primiparity	Yes	100 (78.74%)	138 (83.13%)	0.34
	Delivery mode	Vaginal delivery	95 (74.80%)	85 (51.20%)	< 0.01 *
	Gestational complication	Hypertensive disorder	10 (7.87%)	25 (15.06%)	0.06
		Diabetes	18 (14.17%)	38 (22.89%)	0.06
		Hypothyroidism	14 (11.02%)	11 (6.63%)	0.18
		Other	24 (18.90%)	27 (16.27%)	0.56
	Placental abruption	Yes	1 (0.08%)	6 (3.61%)	0.12
	Fetal distress	Yes	87 (68.50%)	124 (74.70%)	0.24
	Premature rupture of membrane	Yes	35 (27.56%)	34 (20.48%)	0.16
Neonatal baseline	Gender	Male	84 (66.14%)	91 (54.82%)	0.05
	Birth weight (g)		3390.00 (3125.00, 3740.00)	3395.00 (3077.50, 3652.50)	0.41
	Birth length (cm)		50.00 (50.00, 51.00)	50.00 (50.00, 51.00)	0.15
	Severe perinatal asphyxia		13 (10.24%)	8 (4.82%)	0.08
	Fluid intake (mL)		238.30 (196.70, 282.00)	233.95 (194.00, 286.25)	0.85
	Fluid intake/birth weight		68.42 (56.55, 83.51)	70.53 (57.62, 85.48)	0.56
	Urinary volume (mL) urinary volume		50.00 (40.00, 65.00)	116.00 (99.75, 150.00)	< 0.01 *
	Urinary output (mL/kg/h)		0.65 (0.49, 0.82)	1.46 (1.21, 1.77)	< 0.01 *

Categorical and continuous variables are presented as frequency or median (IQR), respectively. * Statistically significant difference (p < 0.05)

### Initial arterial blood gas linked to oliguria during the first 24 h of life in term neonates with asphyxia

Univariate logistic regression analysis (Table 2) was conducted to reveal blood gas-related risk factors for oliguria during the first 24 h after birth in term neonates with PA. It showed that lactate levels (p < 0.01; odds ratio (OR) 1.16; 95% confidence interval (CI) 1.07–1.24) were significantly associated with an increased risk of oliguria, while PCO_2_ (p < 0.01; OR 1.99; 95%CI 1.77–2.24), BE (p < 0.01; OR 1.08; 95%CI 1.03–1.14), and HCO_3_^−^ (p = 0.01; OR 0.92; 95%CI 0.86–0.98) were significantly inversely associated with oliguria in full-term neonates with PA at 24 h of life.

**Table 2:** Peripheral arterial blood gas related risk factors of oliguria in term neonates with perinatal asphyxia within the first 24 h of life

Parameters	UOP < 1.0 ml/kg/h			Univariate logistic regression		Multivariate logistic regression	
	Yes *N* = 127	No *N* = 166	*P*- value	OR (95% CI)	*P*- value	OR (95% CI)	*P*- value
Lactate (mmol/L)	7.40 (5.40, 9.60)	6.15 (4.78, 8.00)	0.00 *	1.16 (1.07–1.24)	0.00 *	1.19 (1.04–1.35)	0.01 *
pH	7.23 (7.16, 7.30)	7.24 (7.19, 7.29)	0.31	1.23 (0.55–2.74)	0.62	1.40 (0.29–6.72)	0.68
PCO_2_ (mmHg)	37.00 (33.00,44.00)	42 .00(35.00, 47.00)	0.01 *	0.98 (0.95–1.00)	0.03 *	0.99 (0.96–1.02)	0.41
PO_2_ (mmHg)	70.00 (56.00, 83.00)	64.50 (50.00, 82.00)	0.08	1.01 (1.00–1.02)	0.11	1.00 (0.99–1.01)	0.86
BE (mmol/L)	–10.30 (–7.90, –14.30)	–9.15 (–6.88, –11.90)	0.01 *	1.08 (1.03–1.14)	0.00 *	0.95 (0.84–1.07)	0.38
HCO_3_^–^ (mmol/L)	16.80 (13.80, 19.30)	17.60 (15.58, 19.70)	0.02 *	0.92 (0.86–0.98)	0.01 *	0.97 (0.88–1.06)	0.49

Categorical and continuous variables are presented as frequency or median (IQR), respectively. * Statistically significant difference (*p* < 0.05)

*BE* base excess, *CI* confidence interval, *Lac* lactic acid, *OR* odds ratio, *PCO*_*2*_ partial pressure of carbon dioxide, *PO*_*2*_, partial pressure of oxygen, *UOP* urinary output

The multivariate regression analysis (Table 2) was conducted to reveal the independent risk factors for oliguria; it indicated that only a high lactate level (p = 0.01; OR 1.19; 95%CI 1.04–1.35) in the blood gas analysis after resuscitation was an independent predictor of oliguria during the first 24 h after birth.

### Potential of initial arterial lactate to predict the risk of oliguria in term neonates with PA

**Fig. 2:**
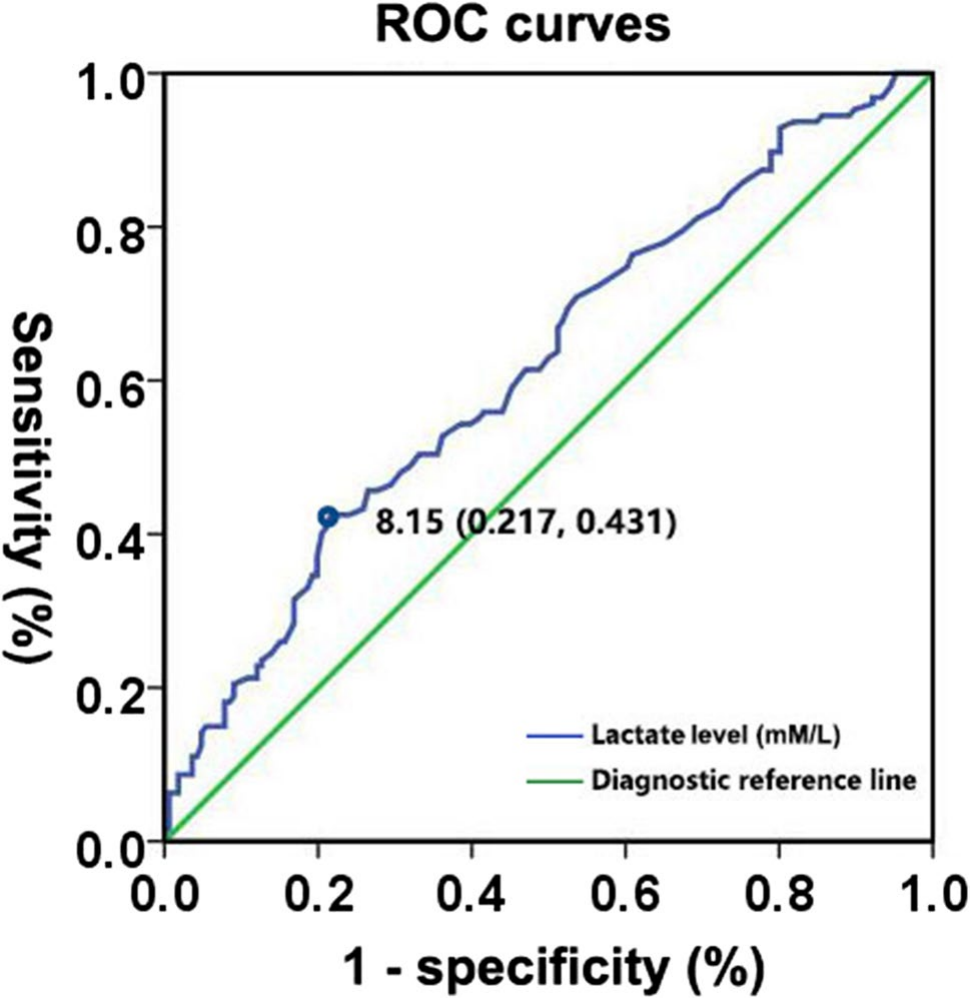
Receiver operating characteristic curves for lactate concentration in arterial blood gas for distinguishing full-term neonates with oliguria from those without oliguria

An ROC curve was constructed to evaluate the potential values of lactate concentration in the peripheral artery gas to distinguish full-term neonates with oliguria from those without oliguria (Fig. 2). Area under the ROC curve (AUC) revealed a moderate discriminatory power of lactate concentration (AUC = 0.62, 95%CI 0.56–0.68, p < 0.01). The lactate threshold (cutoff point) of 8.15 mmol/L was an early indicator of oliguria, with a positive predictive value, negative predictive value, and consistency rate of 59.34% (54/91), 63.86% (129/202), and 62.46% ((54 true positive cases plus 129 true negative cases)/293 total cases), respectively.

Based on the lactate cutoff point, 293 term neonates with PA were categorized into the high lactate level group (≥ 8.15 mmol/L, n = 91) or low lactate level group (< 8.15 mmol/L, n = 202). The UOP and anoxia of neonates in both groups were investigated (Table 3). The high lactate level group showed a higher occurrence of oliguria (54/91 (59.34%) vs. 73/202 (36.14%); χ^2^ = 13.71, p < 0.01), lower UOP (0.89 (0, 0.63) vs. 1.22 (0.37, 2.07) mL/kg/h; z = –3.86, p < 0.01), and lower urinary volume (70.00 (15.00, 125.00) vs. 97.50 (27.50, 167.50) mL; z = –4.39, p < 0.01) than the low lactate level group (Table 3). Furthermore, in the high lactate level group, 84.62% of term neonates with PA experienced intrauterine distress (77/91 (84.62%) vs.134/202 (66.34%); χ^2^ = 10.37, p < 0.01) and 19.78% of neonates experienced severe PA (18/91 (19.78%) vs. 4/202 (1.98%); χ^2^ = 28.53, p < 0.01) (Table 3). The rates of AKI (11/91 (12.09%) vs.10/202 (5.21%); χ^2^ = 0.08, p = 0.03) and mortality (2/91 (2.20%) vs. 0/202 (0%); χ^2^ = 1.82, p = 0.18) in the high lactate level group were higher than those in the low lactate level group (Table 3).

**Table 3:** Clinical characteristics of asphyxiated term neonates with elevated lactate in arterial blood gas

Variables	Lac < 8.15 mmol/L (*n* = 202)	Lac ≥ 8.15 mmol/L (*n* = 91)	*p*- value
Intrauterine distress (%)	134 (66.34%)	77 (84.62%)	0.01 *
Severe perinatal asphyxia (%)	4 (1.98%)	18 (19.78%)	0.01 *
Oliguria (%)	73 (36.14%)	54 (59.34%)	0.01 *
Urinary output (mL/kg/h)	1.22 (0.37, 2.07)	0.89 (0, 0.63)	0.01 *
Urinary volume (mL)	97.50 (27.50, 167.50)	70.00 (15.00, 125.00)	0.01 *
Acute kidney injury (%)	10 (5.21%)	11 (12.09%)	0.03 *
Mortality (%)	0 (0%)	2 (2.20%)	0.18

Categorical and continuous variables are presented as frequency or median (IQR), respectively

*Statistically significant difference (*p* < 0.05)

## Discussion

Asphyxia is a perinatal process of transient hypoxia, or a continuation of intrauterine distress and hypoxia in the fetus during childbirth. Conventional arterial blood gas analysis is internationally recognized as a reliable method for measuring the acid–base balance and hypoxia status of newborns through objective indicators [19]. The pH value reflects the acid–base status of neonates, hypoxia, and hypercapnia in utero. PCO_2_, HCO_3_^−^, and BE values are used to classify acid–base status. Lactate and BE levels reflect the severity of acid–base poisoning [20]. For full-term neonates, renal insufficiency may occur within 24 h of hypoxia and ischemia [9, 10]. To the best of our knowledge, few studies have investigated the predictive potential of blood gases and lactate levels for renal function in newborns with asphyxia. The present study investigated this in 293 term neonates with asphyxia from 9216 hospitalized newborns within a 4-year period.

Early kidney function and UOP monitoring after resuscitation in neonates with asphyxia have always been a focus of attention for NICU medical staff. A considerable number of studies indicate that oliguria is one of the key indicators of AKI in the neonatal population [1, 3, 7]. In a study conducted by Gonzalez et al., the occurrence of oliguria for one hour or more showed fair predictive ability for creatinine-defined AKI (AUC = 0.75). Oliguria lasting for more than 4 h provided the best discrimination, with a 52% sensitivity and 86% specificity [7]. Oliguria is also an early predictor for adverse prognoses in survivors of critical diseases [7, 8]. Early detection of oliguria in term neonates with asphyxia within 24 h of life can provide useful information for timely intervention or treatment and directly impacts the prognosis of neonates. Therefore, it is crucial to identify rapid indicators for predicting oliguria as early as possible. Perinatal medicine researchers have shown considerable interest in the clinical value of arterial blood gas analysis [12]. Our study’s findings provide insight on the link between two conventional parameters in term neonates with PA: the arterial blood gas immediately after resuscitation and oliguria during the first 24 h of life. We preliminarily explored the clinical predictive value of early peripheral arterial blood gas analysis after resuscitation at the first 24 h after birth. We found that a higher lactate concentration was an independent risk factor for PA-complicated oliguria in full-term neonates. Even higher peripheral artery lactate concentrations provided a stepwise increase in specificity when predicting oliguria in full-term neonates with PA. In this study, however, early pH values in the peripheral arterial blood gas after resuscitation did not correlate with oliguria after PA within the first 24 h after birth. The BE value was related but not identified as an independent risk factor for oliguria after PA. After resuscitation, newborns with PA are often given various treatments such as oxygen or bicarbonate. As reported, compared with the pH and BE values, lactate levels varied less when bicarbonate was used [9]. Like previous research data from adult patients [21, 22], early peripheral artery lactate concentration after resuscitation could reflect the kidney function status of full-term neonates with PA during the first 24 h of the life.

An elevated peripheral artery lactate level 0.5–1 h after birth exhibited a moderate predictive value (AUC = 0.619) for oliguria in full-term neonates with asphyxia within the first 24 h of life. The lactate threshold (cutoff point) of 8.15 mmol/L immediately after resuscitation was an early indicator of oliguria, with a positive predictive value, negative predictive value, and consistency rate of 59.34%, 63.86%, and 62.46%, respectively. Following previous studies conducted in adult patients, the predictive value of lactate levels for detecting kidney function status and prognosis has recently gained increasing attention from the medical community. In a prospective observational study involving 1498 adult patients [21], serum lactate levels also demonstrated a moderate predictive value (AUC = 0.633) for severe AKI. In contrast, a serum lactate and cystatin C biomarker combination performed well in predicting severe AKI (AUC = 0.907). Another trial, which included 131 adults with septic AKI requiring kidney replacement therapy, suggested that lactic acid levels were an independent predictor of death [22]. After further observation and analysis, we found that the rates of severe asphyxia, intrauterine distress, AKI, and mortality were higher in term neonates with asphyxia and high lactate levels (≥ 8.15 mmol/L) immediately after resuscitation than in those with low lactate levels. Notably, the incidence of severe asphyxia varied by nearly ten times (19.78 vs. 1.98%) between the two groups of patients. Therefore, monitoring UOP during the first 24 h of life is particularly important for term infants with asphyxia and lactate levels greater than 8.15 at 0.5–1 h after resuscitation, especially for those with a history of intrauterine distress or severe asphyxia.

This study has several strengths and limitations. It explored postnatal oliguria in a neonatal population with asphyxia and confirmed that elevated peripheral artery lactate levels 0.5–1 h after birth are an early and fast predictor of oliguria in neonates with asphyxia within the first 24 h after birth. This finding can help reduce the risk of developing AKI within the first 72 h after birth and encourages timely intervention by neonatologists to improve prognosis. The research was conducted at a single center, with guaranteed standard input and timely treatment given to all newborns by the same medical team, minimizing the variation between different medical groups. Blood gas, lactate, and oliguria levels of the study participants are easily accessible indicators in clinical practice, making the research results applicable in clinical practice. The urine volume is a noninvasive, readily available clinical observation indicator, especially in the early neonatal stages, that is satisfactory for newborn population monitoring. However, this study also has some limitations. This was a retrospective study, and the blood gas analysis data was only collected once, lacking dynamic observation of blood gas data at multiple time points. This study did not explore the time point with a higher diagnostic value for lactate concentration in blood gas. Furthermore, the threshold value for lactate in blood gas, which allows an optimal relationship between sensitivity and specificity in predicting the risk of oliguria, should be precisely determined in future, large, multicenter studies. Additionally, future research should consider combining other fast indicators in routine examinations upon admission to improve the sensitivity of lactate values in peripheral arterial blood gas analysis for predicting the risk of oliguria in term neonates with asphyxia.

## Conclusion

In conclusion, the link between the lactate concentration in peripheral artery gas immediately after resuscitation and oliguria occurring during the first 24 h of life provides new insight regarding the early, rapid evaluation of the UOP of full-term infants with asphyxia in the NICU. The UOP of term infants with a lactate value ≥ 8.15 mmol/L after asphyxia resuscitation should be carefully monitored, especially in neonates with a history of intrauterine distress or severe asphyxia. Meticulously monitoring UOP will allow this vulnerable population to receive early, tailored fluid management and medical intervention, reducing the possibility of poor prognosis.

### Supplementary Information

Below is the link to the electronic supplementary material.

Graphical Abstract (PPTX 146 KB)

## Data Availability

The datasets generated for this study are available on request to the corresponding author.

## Abbreviations

AKI: Acute kidney injury
AUC: Areas under the receiver operating characteristic curve
BE: Base excess
CI: Confidence interval
HCO_3_^−^: Bicarbonate radical
NICU: Neonatal intensive care unit
OR: Odds ratio
PA: Perinatal asphyxia
PCO_2_: Partial pressure of carbon dioxide
PO_2_: Partial pressure of oxygen
ROC: Receiver operating characteristic
UOP: Urine output

## References

[1] Bezerra CT, Vaz Cunha LC, Libório AB (2013). Defining reduced urine output in neonatal ICU: importance for mortality and acute kidney injury classification. Nephrol Dial Transplant.

[2] Fang F, Hu X, Dai X, Wang S, Bai Z, Chen J, Pan J, Li X, Wang J, Li Y (2018). Subclinical acute kidney injury is associated with adverse outcomes in critically ill neonates and children. Crit Care.

[3] Goldstein SL (2020). Urine output assessment in acute kidney injury: the cheapest and most impactful biomarker. Front Pediatr.

[4] Ricci Z, Ronco C (2013). Neonatal RIFLE. Nephrol Dial Transplant.

[5] De Mul A, Parvex P, Héneau A, Biran V, Poncet A, Baud O, Saint-Faust M, Wilhelm-Bals A (2022). Urine output monitoring for the diagnosis of early-onset acute kidney injury in very preterm infants. Clin J Am Soc Nephrol.

[6] Zappitelli M, Ambalavanan N, Askenazi DJ, Moxey-Mims MM, Kimmel PL, Star RA, Abitbol CL, Brophy PD, Hidalgo G, Hanna M, Morgan CM, Raju TNK, Ray P, Reyes-Bou Z, Roushdi A, Goldstein SL (2017). Developing a neonatal acute kidney injury research definition: a report from the NIDDK neonatal AKI workshop. Pediatr Res.

[7] Gonzalez F, Vincent F (2012). Oliguria as predictive biomarker of acute kidney injury in critically ill patients. Minerva Anestesiol.

[8] Macedo E, Malhotra R, Bouchard J, Wynn SK, Mehta RL (2011). Oliguria is an early predictor of higher mortality in critically ill patients. Kidney Int.

[9] Simovic A, Stojkovic A, Savic D, Milovanovic DR (2015). Can a single lactate value predict adverse outcome in critically ill newborn?. Bratisl Lek Listy.

[10] Das MK, Ali MA, Latif T, Islam MN, Hossain MA, Moniruzzaman MM, Oliullah M, Haque SA, Gosh AK (2018). Comparison of serum electrolytes abnormality and renal function status in asphyxiated and normal baby in a tertiary level hospital. Mymensingh Med J.

[11] Alaro D, Bashir A, Musoke R, Wanaiana L (2014). Prevalence and outcomes of acute kidney injury in term neonates with perinatal asphyxia. Afr Health Sci.

[12] Cai Y, Zhang X, Wu X, Liu H, Qi L, Liu X (2022). The value of umbilical artery blood gas analysis in the diagnosis and prognosis evaluation of fetal distress. Am J Transl Res.

[13] Younus J, Hayat S, Haroon F, Irfan Waheed KA, Khan MQ, Khalid MU (2020). Correlation of severity of metabolic acidosis at admission and outcome in asphyxiated neonates. J Ayub Med Coll Abbottabad.

[14] Gomella TL, Cunningham MD, Eyal FG (2013). Neonatology: management, procedures, on-call problems, diseases, and drugs.

[15] Shao XM, Ye HM, Qiu XS (2019). Practice of neonatology.

[16] Neonatal Resuscitation Group, Subspecialty Group of Perinatal Medicine, Chinese Medical Association (2016). Experts' consensus on the diagnosis of neonatal asphyxia. Chin J Perinat Med.

[17] Hua KQ, Feng YJ (2013). Practice of obstetrics gynecology.

[18] Borowska-Matwiejczuk K, Lemancewicz A, Tarasów E, Urban J, Urban R, Walecki J, Kubas B (2003). Assessment of fetal distress based on magnetic resonance examinations: preliminary report. Acad Radiol.

[19] Mikkelsen SH, Olsen J, Bech BH, Wu C, Liew Z, Gissler M, Obel C, Arah O (2017). Birth asphyxia measured by the pH value of the umbilical cord blood may predict an increased risk of attention deficit hyperactivity disorder. Acta Paediatr.

[20] Giovannini N, Crippa BL, Denaro E, Raffaeli G, Cortesi V, Consonni D, Cetera GE, Parazzini F, Ferrazzi E, Mosca F, Ghirardello S (2020). The effect of delayed umbilical cord clamping on cord blood gas analysis in vaginal and caesarean-delivered term newborns without fetal distress: a prospective observational study. BJOG.

[21] Hou Y, Deng Y, Hu L, He L, Yao F, Wang Y, Deng J, Xu J, Wang Y, Xu F, Chen C (2021). Assessment of 17 clinically available renal biomarkers to predict acute kidney injury in critically ill patients. J Transl Int Med.

[22] Fukuda M, Fukami K, Nabeta M, Hirayu N, Takasu O (2023). Association of baseline renal function with mortality in patients with sepsis requiring continuous renal replacement therapy for acute kidney injury: a single-center retrospective study. Blood Purif.

