# Supplementary material for: Initial lactate levels linked to oliguria in term neonates with perinatal asphyxia
Source: Pediatr Nephrol. 2024 Feb 27;39(7):2227–34. doi: 10.1007/s00467-024-06322-8 (PMC11147877; doi:10.1007/s00467-024-06322-8)
Supplement: Supplementary file 1 — Graphical Abstract (PPTX 146 KB) [file 467_2024_6322_MOESM1_ESM.pptx]

## Slide 1
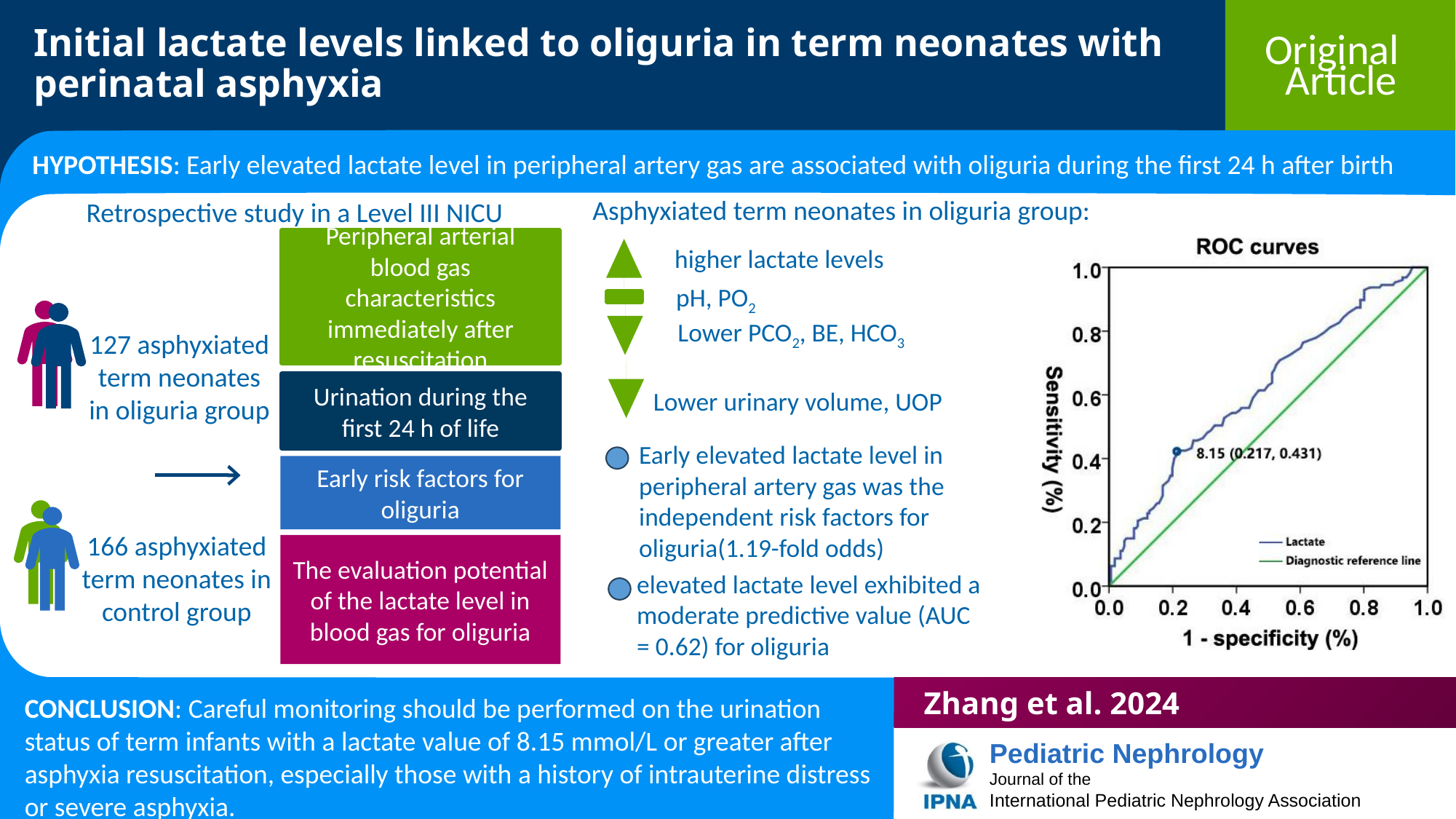

Initial lactate levels linked to oliguria in term neonates with
perinatal asphyxia
HYPOTHESIS: Early elevated lactate level in peripheral artery gas are associated with oliguria during the first 24 h after birth
Asphyxiated term neonates in oliguria group:
Retrospective study in a Level III NICU
Peripheral arterial blood gas characteristics immediately after resuscitation
higher lactate levels
pH, PO2
Lower PCO2, BE, HCO3
127 asphyxiated term neonates in oliguria group
 Urination during the
first 24 h of life
Lower urinary volume, UOP
Early elevated lactate level in peripheral artery gas was the independent risk factors for oliguria(1.19-fold odds)
Early risk factors for oliguria
166 asphyxiated term neonates in control group
The evaluation potential of the lactate level in blood gas for oliguria
elevated lactate level exhibited a moderate predictive value (AUC = 0.62) for oliguria
Zhang et al. 2024
CONCLUSION: Careful monitoring should be performed on the urination status of term infants with a lactate value of 8.15 mmol/L or greater after asphyxia resuscitation, especially those with a history of intrauterine distress or severe asphyxia.
